# The journey is the reward

**DOI:** 10.4103/0019-5545.58885

**Published:** 2010

**Authors:** T. S. Sathyanarayana Rao

**Affiliations:** Department of Psychiatry, JSS University, JSS Medical College Hospital, Ramanuja Road, Mysore - 570 004, India

You have achieved success in your field when you don't know whether what you're doing is work or play.

Warren Beatty.

Quoted in Deccan chronicle

Bengaluru Edition, 19^th^ Oct 2009

What a wonderful year it was! I have picked up the quote above simply because the exhilaration of many positives we had over shadowed all the toil it entails in getting the journal in shape and in time. Before I get to mention the Pubmed Central indexing of our journal, there are a few points to be certainly noted.

1. The journal is getting released each time, in time. The final issue of the last year 51(4) was in web by early December and the New Year Issue, Jan – March 2010 is already in web in the second week of Jan 2010. I will assure you, being certain of continuity in the office, that the April-June issue will be ready by February 2010.
2. The special supplement of Dementia under the guest editorial of Dr. K.S. Shaji is a big hit and is already well quoted.
3. I must thank Dr. Sudheer Kumar, Organizing Secretary, ANCIPS, 2009 at Agra for the timely arrangement made for the publication of abstracts in April supplement.

## INDEXING OF THE JOURNAL

Apart from the many indexing agencies quoted in the general information sheet in the journal, the crowning glory as the indexing of our journal at Pubmed Central. I must thank our president, Dr. E. Mohandas, Immediate Past Presidents Dr. P.C. Shastri and Dr. Indla Ramasubba Reddy and the incoming president Dr. Ajit Avasthi at the outset. Dr. Govind M. Bang, Honarary General Secretary, Dr Asim Kumar Mallick, Honarary Treasurer deserve special mention. I am grateful to our editorial office bearers, Dr. G. Swaminath, Dr. Roy Abraham Kallivayalil, Dr. G. Prasad Rao, Dr. Ajith V. Bhide and Dr. Sandeep Grover, Editorial board members, Journal Committee members, National and International Advisory Board members, the reviewers and the authors who made this possible. Ultimately much of the credit must go to Dr. Sahu and his team at Medknow Publications, Mumbai, who are responsible for this achievement. My sincere thanks also go to Prof Mario Maj, Dr. J. Mezzich and the world Psychiatric Association (WPA), in particular Dr. Helen Hermann, Dr. Jair Mari, Dr. Vikram Patel, Dr. Christian Keiling and others. Very specifically I am grateful to Prof Maj and all in the task force, as well as observer Dr Shekhar Saxena, consultant Prof Norman Sartorius, Anna Engstrom and those in WPA Who have helped including members of the executive committee for support and advice.

The major success of indexing goes to Dr. Sahu and his team. I like to quote his letter Which also makes our responsibilities increase many told:

### From Dr. Sahu, Medknow Publications and Media Pvt Ltd

Dated 11^th^ September 2009

‘We are pleased to inform that the Indian Journal of Psychiatry is now indexed with PubMed. One can search the articles published in IJP from 2009 onwards from PubMed (http://www.ncbi.nlm.nih.gov/sites/entrez?db=pubmedandterm=%22Indian%20J%20Psychiatry% 22 [Journal]). All the articles will get linked to IJP's website in next three to four days.

The full text of IJP is also archived with PMC (http://www.pubmedcentral.nih.gov/tocrender.fcgi?journal=938andaction=archive) and searchable separately from Entrez PubMed. we will provide the back volumes to PMC in a couple of weeks so that all the articles from 2007 of IJP are available in PubMed. Older issues will be added over a few months.

This being one of the important milestones, it is important that we continue to maintain and improve the quality of papers by rigorous peer-review and maintain the publication schedule so as to take the maximum advantage of PubMed indexing. Our next goal would be indexing with Science Citation Index for which both quality of articles and regularity of publication are important.’

## FURTHER IMPROVING OUR VISIBILITY

we want your help in this process. It goes without saying that a researcher contributes to a peer reviewed scholarly journal for gaining the research impact and peer recognition. The internet today provides more opportunities than before to improve the visibility of your research work. There have been some studies to show direct correlation of article downloads to citations received. The journal's website and its bibliographic linking helps get readers. However, additional linking by you would help you to get even higher citations and research impact. You can use a few (or all) of the online tools for this purpose.

1. If your institution has an open archive repository, you can put the PDF of your article in the archive with a link to the article on the journal's website. Check http://www.eprints.org/openaccess/self-faq/#self-archiving to learn more about archiving.
2. Deposit the article in a subject based OAI-PMH compliant repository. You can find subject wise list of repositories from http://www.opendoar.org/find.php and http://opcit.eprints.org/explorearchives.shtml#disciplinary.
3. Link your paper from as many websites as possible using citation and social book marking tools such as GetCited, CiteULike, Connotea, Zotero, etc. The URLs for registering with few of these sites are
  http://www.getcited.org/add/
  http://www.citeulike.org/register
  http://www.connotea.org/register
  http://www.indexcopernicus.com/info.php?id=1
  http://www.zotero.org/
  http://www.stumbleupon.com/sign-up.php?pre2=hp-join
4. Link the article from an appropriate topic in the wikipedia e.g. http://en.wikipedia.org/wiki/Acanthamoeba—keratitis#References [Ref. 5] or http://en.wikipedia.org/wiki/Hallermann-Streiff—syndrome#References.
5. As an author you can also deposit your paper with the NLM's PubMedCentral, if you have received an NIH grant. Use the myNCBI link on http://www.nihms.nih.gov/db/sub.cgi to submit your paper.
6. Link the paper from your personal / institution web pages.

## THE FUTURE

The future Is very encouraging. A significant overhaul of our boards is on the agenda. The steps are being taken to strengthen the editorial office further so also the clearing of the backlog. You might have already noticed the increase in the number of pages of each issue, being around 100 pages. The new initiatives in the form of “Annotations of Indian Psychiatry” and “The ICONS of Indian Psychiatry” are Slated for the release in January, coinciding with ANCIPS 2010 at Jaipur. I am confident that they will go a long way in enhancing our impact factor at Science Citation Index.

Ultimately, I have the satisfaction of mission accomplished. I am confident the New Year will be the beginning of an arduous travel ahead for the much more to be achieved. Well begun is war already half won! To repeat, you can certainly expect more from us!

Personally, and on behalf of the editorial team, I wish you a happy reading and a happy new year.

**Long Live IJP and IPS**

**Dr. T. S. Sathyanarayana Rao**

**10^th^ December 2009**

